# Interpreting peripheral oxygen saturation variability in critical illness: A directional framework adjusted for hypoxia severity

**DOI:** 10.1113/EP093235

**Published:** 2026-02-11

**Authors:** Shuyang Iris Feng, Tope Oyelade, Mudra Ko, Yunkai Zhang, Watjana Lilaonitkul, Thomas B. Williams, Joseph T. Costello, Ali R. Mani

**Affiliations:** ^1^ Network Physiology Lab, UCL Division of Medicine University College London London UK; ^2^ School of Medicine Keele University Staffordshire UK; ^3^ School of Medicine and Dentistry University of Central Lancashire Preston UK; ^4^ Global Business School for Health University College London London UK; ^5^ Extreme Environments Laboratory, School of Psychology, Sport and Health Sciences University of Portsmouth Portsmouth UK; ^6^ Institute for Liver and Digestive Health, UCL Division of Medicine University College London London UK

**Keywords:** acute liver failure, cirrhosis, COPD, extreme environments, pulse oximetry, sample entropy, sepsis, $S_{pO_{2}}$ variability

## Abstract

Peripheral oxygen saturation ($S_{pO_{2}}$) exhibits a complex pattern of fluctuations during hypoxia, which can be quantified using entropy measures. $S_{pO_{2}}$ entropy analysis provides insights into dynamic physiological regulation by non‐invasively reflecting the body's capacity to adapt to internal or external physiological challenges. However, the interpretation of $S_{pO_{2}}$ entropy alone is limited without contextualisation and the degree of physiological challenge encountered (e.g. the severity of hypoxia). This proof‐of‐concept retrospective study analysed continuous 1 Hz $S_{pO_{2}}$ recordings extracted from MIMIC‐III dataset's Intensive Care Unit ICU patients with sepsis (*n* = 164), chronic obstructive pulmonary disease (COPD) (*n* = 58), acute liver failure (ALF) (*n* = 59), or cirrhosis (*n* = 169). Sample entropy was computed directly from raw 20‐min $S_{pO_{2}}$ signals and normalised to mean $S_{pO_{2}}$ using directional parenclitic deviation (δ), derived from a healthy hypoxia‐exposure reference dataset. Cox‐regression models assessed 30‐day ICU mortality. In sepsis, δ was significantly higher in non‐survivors (hazard ratio (HR) = 2.20, *P *< 0.0001) and independently predicted 30‐day mortality (HR = 1.79, *P *< 0.0001). δ was not predictive in the COPD, ALF and cirrhosis cohorts. Unlike other patient groups, the cirrhosis group demonstrated unexpected mean negative δ values, suggesting aberrant regulatory engagement, potentially related to the pathophysiology of hepatopulmonary syndrome. These findings demonstrate that δ provides physiological contexts to entropy‐based $S_{pO_{2}}$ analysis. By linking variability to the severity of hypoxia, this framework enables a more interpretable and a potentially clinically applicable biomarker of systemic regulation in critical illnesses. Future validation across diverse cohorts could support its potential to aid in personalised care within intensive care settings.

## Abstract (highlights)

- **What is the central question of this study?**
  Can peripheral oxygen saturation ($S_{pO_{2}}$) variability, when normalised relative to its mean, provide clinically meaningful insights into physiological regulation and prognosis in critically ill patients?
- **What is the main finding and its importance?**
  Normalising $S_{pO_{2}}$ variability using the parenclitic deviation (δ) from the correlation between $S_{pO_{2}}$ entropy and mean $S_{pO_{2}}$ in healthy subjects provides prognostic insights in sepsis. The direction and size of δ indicate distinct patterns of physiological dysregulation in sepsis, COPD and liver cirrhosis.

## 1 INTRODUCTION

**FIGURE 1:**
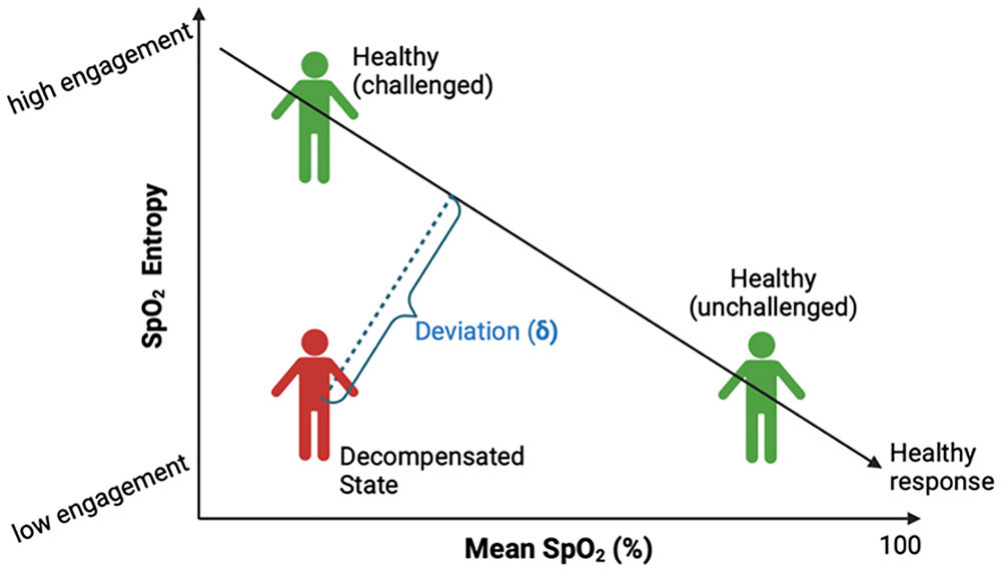
Contextual interpretation of $S_{pO_{2}}$ entropy using physiological challenge. Sample entropy (SampEn) measures the complexity of a time‐series such as $S_{pO_{2}}$. In healthy individuals, entropy increases during an acute hypoxic challenge and decreases in normoxia (Costello et al., 2020). This creates an inverse relationship between $S_{pO_{2}}$ entropy and mean $S_{pO_{2}}$ in a healthy reference population (i.e. black line). However, in critically ill patients, low entropy may reflect either a stable, unchallenged state or a pathologically decompensated one. Parenclitic deviation (δ), shown as the perpendicular distance from a patient's point to the healthy reference trajectory, captures the degree and direction of divergence from expected adaptive behaviour, enabling more meaningful interpretation of OSV patterns.

Peripheral oxygen saturation ($S_{pO_{2}}$) can be measured non‐invasively and has numerous applications in clinical practice as well as in monitoring individuals exposed to extreme environments. Recent studies have indicated that oxygen saturation ($S_{pO_{2}}$) signals exhibit a complex pattern of fluctuations that can be assessed using non‐linear methods such as entropy analysis (Boghal & Mani, 2017; Costello et al., 2020; Morandotti et al., 2025a). Oxygen saturation variability (OSV) analysis has been investigated as a promising biomarker in monitoring patients with critical illness, reflecting the body's ability to dynamically regulate oxygen delivery by active engagement of integrated physiological control mechanisms (Jiang et al., 2021; Gheorghita et al., 2022; Morandotti et al., 2025a). However, a major issue in OSV research is its interpretation, since variability alone is not inherently meaningful, unless it is understood within the context of the system's physiological state and, most importantly, the challenges imposed on it (e.g. hypoxia). For instance, it is expected to see more engagement of cardiorespiratory control mechanisms during hypoxia that leads to increased complexity (and variability) of $S_{pO_{2}}$ signals, while during normoxia the physiological system is not challenged, leading to a stable (and less variable) $S_{pO_{2}}$ signal (Costello et all., 2020; Jiang et al., 2021) (Figure 1).

Sample entropy (SampEn) measures the regularity and complexity of a time series such as $S_{pO_{2}}$. In OSV analysis, higher entropy suggests greater irregularity, which in some cases reflects a system's ability to adapt to physiological stress, while lower entropy suggests more regularity, which could be either stable due to minimum physiological challenge or a rigid, dysfunctional system that cannot adapt to physiological challenges (Jiang et al., 2021; Morandotti et al., 2026b). This dual interpretation poses a key problem: when should low entropy be considered as a sign of decompensated physiological adaptation? To answer this, it is essential to consider physiological challenges, which is a perturbation that demands a response from the body. In health, this adaptive rise in entropy during hypoxic challenge contrasts with the blunted response often observed in critical illness (Gheorghita et al., 2022). Some may show low entropy due to exhausted regulatory capacity, but others may show low entropy due to minimum hypoxic challenge. Therefore, $S_{pO_{2}}$ entropy should not be interpreted independently but rather in conjunction with mean $S_{pO_{2}}$ and within the context of a superimposed physiological challenge or stress (e.g., acute hypoxia).

A similar issue has been recognised in heart rate variability (HRV) research, where HRV depends not just on autonomic function but also on basal mean heart rate (Monfredi et al., 2014). Studies have shown that for HRV to be interpreted meaningfully, it must be corrected for basal heart rate as a healthy HRV value is different for different ranges of basal heart rate in health and disease (Monfredi et al., 2014; Boghal et al., 2019). Indeed, while OSV is dependent on mean $S_{pO_{2}}$, there has been no established normalisation framework to account for this dependency. Without such correction, variability metrics alone may not provide meaningful mechanistic insights.

Further, current clinical assessments of disease heavily rely on simplistic scoring systems such as the Sequential Organ Failure Assessment (SOFA) score for sepsis and the Model for End‐Stage Liver Disease (MELD) score for cirrhosis. These metrics capture systemic dysfunction through laboratory and physiological parameters considered in isolation, but they do not account for the pattern of fluctuations (variability) in the signals, which contains information about the state of physiological control. Thus, incorporating variability metrics like SampEn or standard deviation may complement and enhance traditional risk stratifications.

In this study, we quantify $S_{pO_{2}}$ entropy relative to mean $S_{pO_{2}}$ using directional parenclitic deviation, a graph‐based approach that captures deviations from expected behaviour (Zanin et al., 2014; Oyelade et al., 2023). This approach involves measurement of the deviation (δ) of each participant's signals from the relationship between $S_{pO_{2}}$ mean and $S_{pO_{2}}$ entropy of a reference population that represents healthy individuals’ response to hypoxia (Figure 1). Conceptually, it represents the shortest perpendicular distance from a patient's data point to the healthy reference line on a two‐dimensional plane (see Figure 1 for an overview).

To establish a novel methodological framework for normalising $S_{pO_{2}}$ entropy relative to mean $S_{pO_{2}}$, this study uses sepsis, chronic obstructive pulmonary disease (COPD), acute liver failure (ALF) and liver cirrhosis as examples of critical illnesses that each involve distinct forms of abnormal tissue oxygenation and network‐level dysfunction. Although these conditions are heterogeneous, they share a common feature of affecting multiple organ systems (Ito et al., 2025; Barnes & Celli, 2009; Oyelade et al., 2024a and 2024b). While data from these cohorts serve as case examples, the overarching aim is to establish a proof‐of‐concept for a generalizable methodology for $S_{pO_{2}}$ variability analysis, bridging the importance gap between raw variability metrics and meaningful physiological interpretation and clinical translation.

The primary hypothesis is that critically ill patients would demonstrate systematic deviations from the healthy $S_{pO_{2}}$ mean‐entropy relationship, and that the direction and magnitude of this deviation (δ) would reflect distinct patterns of physiological dysregulation across disease types. Specifically, it is hypothesised that in all four groups (sepsis, COPD, ALF and decompensated cirrhosis), impaired adaptability would lead to suppressed entropy and therefore positive δ. Further, this study investigates whether δ is positive or negative, the differences between survivors and non‐survivors, and the ability of δ to predict 30‐day mortality alongside conventional clinical scores.

## 2 METHODS

### 2.1 Ethical approval

MIMIC‐III is publicly available to researchers under a data use agreement. The data have been deidentified according to HIPAA standards, and the project was approved by the Institutional Review Boards of Beth Israel Deaconess Medical Center and MIT (IRB protocol nos. 2001P001699/14 and 0403000206) and the study conformed to the standards set by the *Declaration of Helsinki*, except for registration in a database. Individual patient consent was waived by the ethics committee as the project did not affect clinical care, and all protected health information was deidentified. The authors involved in data extraction completed mandatory online ethics training at MIT and were credentialled (IDs 10304625 and 48067739). The data used for the reference group of healthy volunteers exposed to normobaric hypoxia were obtained from a study approved by the University of Portsmouth Ethics Committee (project number 2017–025). All participants provided their written informed consent before taking part in this study and the study conformed to the standards set by the *Declaration of Helsinki*, except for registration in a database. The data used for healthy volunteers without hypoxic challenge were recorded following approval of the UCL Ethics committee (ID: 10525/001). All participants provided informed consent, and the study was conducted in accordance with the standards set by the *Declaration of Helsinki*, except for registration in a database.

### 2.2 Reference dataset

**FIGURE 2:**
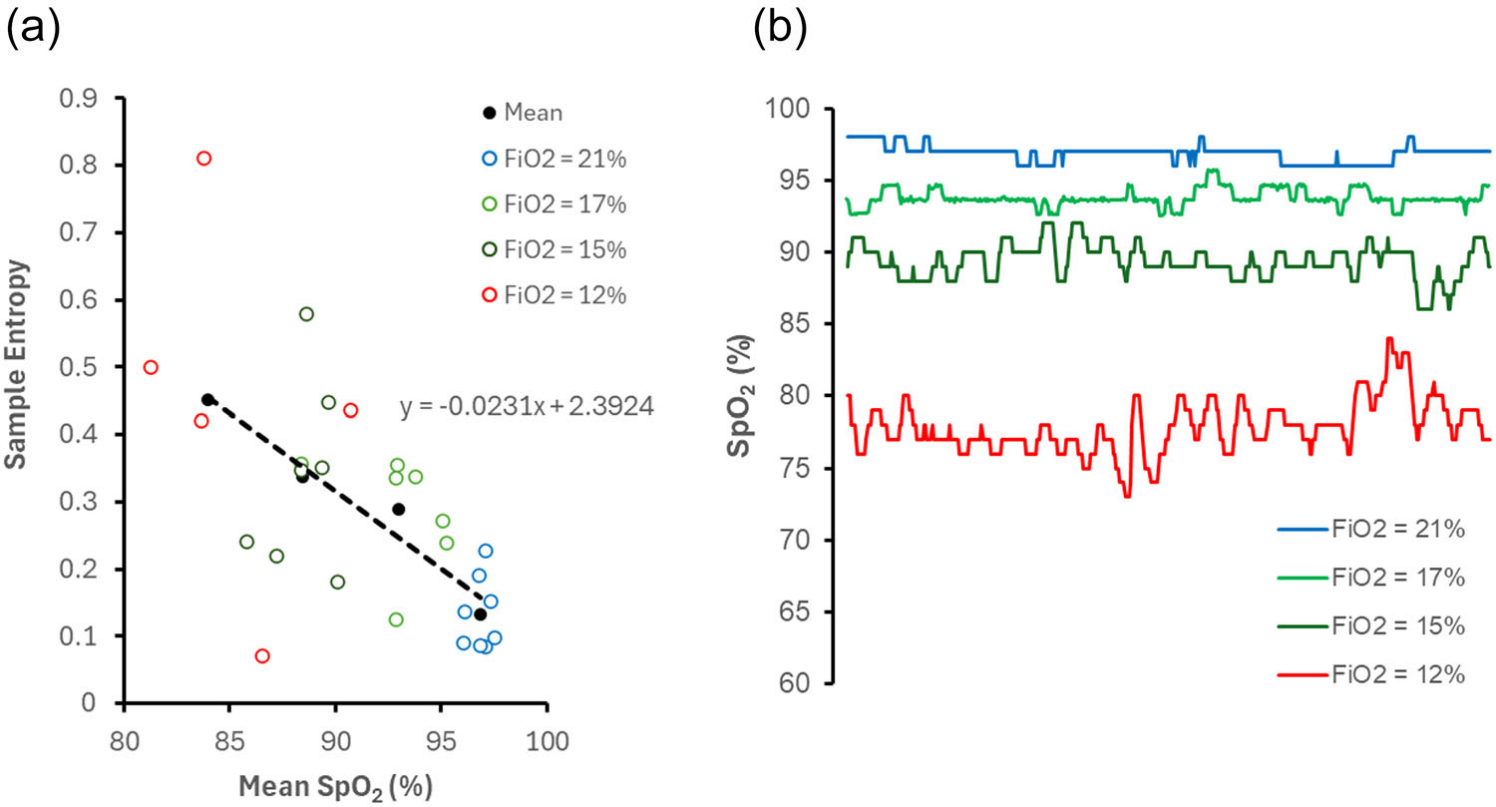
(a) Relationship between $S_{pO_{2}}$ mean and entropy in healthy participants exposed to different inspired oxygen concentrations. A strong inverse linear relationship (*R* ^2^ = 0.999) indicates that $S_{pO_{2}}$ entropy increases as oxygen availability decreases, consistent with greater physiological engagement under hypoxic stress. This linear trend was used to establish the normative reference for subsequent parenclitic deviation analyses. (b) Representative $S_{pO_{2}}$ time‐series from a healthy volunteer under graded hypoxic challenge. A representative $S_{pO_{2}}$ trace (sampled at 1 Hz) from a healthy volunteer undergoing normobaric hypoxia. The participant was sequentially exposed to decreasing inspired oxygen concentrations, resulting in progressively lower mean $S_{pO_{2}}$ levels. The increasing signal irregularity with hypoxia demonstrates greater $S_{pO_{2}}$ entropy, supporting the use of this dataset as a reference model for parenclitic deviation analysis. This trace is shown for descriptive purposes only and is not intended to represent group‐level trends.

To establish a physiological reference for the responsiveness of healthy individuals to hypoxia, raw data from a human normobaric hypoxia experiment were used (Costello et al., 2020). In that study, a total of 12 healthy participants were included in the reference analysis for the fraction of inspired oxygen ($F_{iO_{2}}$) level at 21%, 17% and 14.5%, and 11 participants for $F_{iO_{2}}$ at 12%, following 45‐min exposures at each level. In brief, during each experimental trial, participants were exposed to normobaric hypoxia in a purpose‐built hypoxic chamber (Sporting Edge, Sherfield on Loddon, UK) while at rest. Continuous $S_{pO_{2}}$ recordings were collected at 1 Hz (one sample per second) during each exposure level using a Nonin Medical, Inc., (Plymouth, MN, USA) pulse oximeter. The final 8‐min segment from each condition was extracted to approximate a more stable hypoxic state and to simulate sustained physiological challenge. Mean $S_{pO_{2}}$ and sample entropy were calculated separately for each participant and used to examine the relationship between $S_{pO_{2}}$ mean and entropy in the reference population. The linear relationship between $S_{pO_{2}}$ mean and entropy was obtained using regression analysis (Figure 2a,b). This reference data was collected at the University of Portsmouth, as described by Costello et al. (2020).

### 2.3 ICU patient cohort (MIMIC‐III)

This retrospective study used data from the MIMIC‐III Waveform Database Matched Subset (Johnson et al., 2016). Four patient groups were analysed: sepsis, COPD, ALF and cirrhosis. Patients were included if they had a single ICU stay, were over 18 years old, and had at least 10 min of continuous, noise‐free $S_{pO_{2}}$ waveform data recorded at a sampling rate of 1 Hz. Waveform data, including the $S_{pO_{2}}$ signal, were extracted from the earliest available numeric time‐series during the first day of each patient's initial ICU admission. The SOFA score was calculated for the same day from which the waveform data were obtained. Noise‐free time‐series were defined as time‐series containing a valid time‐stamped value for every second in the waveform database. Consequently, the included time‐series had no missing values, and no imputation was necessary.

For the sepsis group, adult patients were identified according to Sepsis‐3 criteria (Singer et al., 2016): a SOFA score increase of ≥2 points and suspicion of infection, defined by the temporal proximity of antibiotic administration and body fluid culture acquisition. From an initial 179 patient records, 15 lacked 30‐day ICU survival data, resulting in 164 patients being included in the final survival analysis. This sepsis patient recruitment and data acquisition mirror those used in Gheorghita et al. (2022).

For the COPD group, this cohort study retrospectively utilises data from the MIMIC‐III Clinical Database. To identify patients with acute exacerbation of COPD, the corresponding International Classification of Diseases (ICD‐9 code) 49121 was used. The inclusion criteria for adult patients (aged 18 or older) were those who were admitted to the ICU primarily due to acute exacerbations of COPD. A total of 58 patients were included in the final survival analysis.

For the ALF and patients with cirrhosis group, adult patients were selected based on a diagnosis of ALF and cirrhosis, respectively, according to ICD‐9 codes (570 and 571) and the availability of continuous $S_{pO_{2}}$ waveform data sampled at 1 Hz, along with corresponding clinical, laboratory and demographic information. Inclusion criteria for the ALF group were patients aged 18 or older with paracetamol‐induced ALF at ICU admission (see Oyelade et al., 2024b for patient recruitment). For both ALF and cirrhosis patients, those with incomplete clinical data, missing follow‐up or mortality records were excluded. This resulted in a total of 59 ALF and 169 cirrhosis patients included in the final survival analysis.

All patients from the sepsis, COPD or cirrhosis groups were analysed with 20‐min $S_{pO_{2}}$ segments. The duration of the $S_{pO_{2}}$ time‐series was 10 min for patients with ALF, due to limitations in longer data availability. Survival status was defined as alive or deceased at 30 days after ICU admission. In the cirrhosis group, patients who underwent liver transplantation during their ICU stay were labelled as non‐survivors, based on the rationale that transplantation indicates irreversible end‐stage disease and that these patients could not survive without it (Bhogal et al., 2019). $S_{pO_{2}}$ signals recorded (sample rate 1 Hz, duration 20 min) from healthy volunteers were also used as a comparator in this study using a Nonin pulse oximeter. The data were recorded following approval of the UCL Ethics committee (10525/001).

### 2.4 $S_{pO_{2}}$ entropy calculation

Sample entropy (SampEn) was used to quantify the irregularity of $S_{pO_{2}}$ fluctuations. SampEn is a statistical measure used to quantify the amount of unpredictability in time‐series data. Specifically, it measures the negative natural logarithm of the probability that two sequences that are similar for m points (with a degree of tolerance r) remain similar at the next point *m* + 1 (Richman & Moorman, 2000).

SampEn was computed in MATLAB (MathWorks, Natick, MA, USA) using standard parameters: window size of *m* = 2 and degree of tolerance *r* = 0.2 according to Bhogal & Mani (2017). In addition to single‐scale entropy, multiscale entropy (MSE) was also calculated to distinguish between complex and random time series (Bhogal & Mani, 2017; Costa et al., 2002). This method involves coarse‐graining the original time‐series over five time scales (non‐overlapping windows) and computing SampEn at each scale; the values were then averaged to obtain an overall measure of complexity.

### 2.5 Parenclitic deviation (δ) calculation

To assess how patient entropy patterns diverged from those of the healthy reference, a parenclitic deviation framework was applied (Zanin et al., 2014; Ito et al., 2025). This approach evaluates the vertical deviation of a patient's entropy value from the best‐fit regression line derived from the reference dataset calculated from healthy individuals in response to hypoxia (i.e., entropy vs. $S_{pO_{2}}$ mean across different $F_{iO_{2}}$ levels).

$$\delta =\frac{m\times x-y+b}{\sqrt{m^{2}+1}}$$

In the formula, x is mean $S_{pO_{2}}$, and y is SampEn of $S_{pO_{2}}$. The healthy reference line is derived from linear regression on data collected from healthy participants under graded hypoxia, where m is the slope of the reference regression line and b is the *y*‐intercept (Figure 2).

Importantly, absolute values were not applied to calculate these deviations (distance) which is different from the conventional parenclitic calculation. This was based on the hypothesis that the direction of deviation (whether above or below the healthy adaptive pattern) has distinct physiological implications, and thus preserving this directionality may yield more meaningful clinical insights than traditional parenclitic distance‐based approaches.

### 2.6 Statistical analysis

Descriptive statistics were presented as means ± standard deviation for continuous variables and counts for categorical variables. Group comparisons between survivors and non‐survivors were performed using an independent‐samples Student's *t*‐test, one‐way ANOVA or two‐way ANOVA, as appropriate. To evaluate the prognostic value of $S_{pO_{2}}$ variability metrics, Cox proportional hazards regression was used with 30‐day ICU survival as the outcome. The proportional hazards assumption was tested prior to Cox regression analysis. Variables included in the multivariable Cox regression models were selected based on clinical relevance (e.g. ventilation status and SOFA) and prior literature (e.g. $S_{pO_{2}}$ entropy). Univariable Cox analyses were initially performed, and variables with *P* ≤ 0.05 were considered for inclusion. All continuous predictors were standardized (*Z*‐scored) prior to Cox regression to enable comparability of effect sizes. Analyses were performed using IBM SPSS Statistics (IBM Corp., Armonk, NY, USA) and GraphPad Prism (GraphPad Software, Boston, MA, USA), with significance defined as *P* ≤ 0.05.

## 3 RESULTS

### 3.1 Cohort characteristics

A total of 450 ICU patients were included in this study including 321 males (57.52%) with overall mean age of 57.4 ± 18.5 years. Summary descriptive statistics of each cohort, including mean $S_{pO_{2}}$, entropy values and δ, are presented in Table 1. Sex distributions were relatively balanced across groups.

**TABLE 1:** Summary of demographic and signal characteristics of included individuals across healthy and disease groups.

	Healthy (*n* = 108)	Sepsis (*n* = 164)	COPD (*n* = 58)	ALF (*n* = 59)	Cirrhosis (*n* = 169)
Age	39 ± 16	67 ± 17	70 ± 11	53 ± 18	57 ± 13
Sex (M/F)	51/57	94/70	35/23	36/23	105/64
Survivor/non‐survivor	N/A	130/34	37/21	29/30	89/80
$S_{pO_{2}}$ mean	97.7 ± 1.3	97.1 ± 3.5	95.7 ± 3.4	96.7 ± 3.3	97. 0 ± 3.0
$S_{pO_{2}}$ entropy	0.115 ± 0.064	0.097 ± 0.086	0.136 ± 0.135	0.172 ± 0.222	0.191 ± 0.248
Parenclitic deviation (δ)	0.020 ± 0.051	0.053 ± 0.097	0.046 ± 0.136	−0.013 ± 0.217	−0.039 ± 0.243

COPD, Chronic obstructive pulmonary disease; ALF, acute liver failure.

Mean age was significantly different (one‐way ANOVA, *P* < 0.0001) between groups, with COPD and sepsis groups being older on average and the ALF group slightly younger. Sex distribution did not differ significantly between cohorts (χ^2^ *P* = 0.470).

### 3.2 Differences in parenclitic deviations

Mean parenclitic deviation (δ) was compared between survivors and non‐survivors using unpaired *t*‐tests. As shown in Table 2, only the sepsis group showed a statistically significant difference in δ between survivors and non‐survivors, with non‐survivors exhibiting significantly higher δ (0.113 ± 0.126 vs 0.0391 ± 0.0827, *P* < 0.001, *n* = 164), indicating greater deviation from the healthy entropy and $S_{pO_{2}}$ correlation. No significant differences were observed in the COPD, ALF and cirrhosis groups (Table 2).

**TABLE 2:** Comparison of signal features and clinical severity scores between survivors and non‐survivors across disease groups.

	Survivors	Non‐survivors	*P*
Sepsis			
*n*	130	34	
$S_{pO_{2}}$ mean	97.4 ± 2.2	95.9 ± 6.3	0.183
$S_{pO_{2}}$ entropy	0.110 ± 0.0860	0.0745 ± 0.0834	**0.045**
δ	0.0391 ± 0.0827	0.113 ± 0.126	**<0.0001**
SOFA	4.10 ± 2.261	6.82 ± 4.145	**<0.001**
COPD			
*n*	37	21	
$S_{pO_{2}}$ mean	95.3 ± 3.9	96.3 ± 2.4	0.252
$S_{pO_{2}}$ entropy	0.130 ± 0.127	0.146 ± 0.152	0.678
δ	0.0592 ± 0.128	0.0217 ± 0.148	0.314
SOFA	3.86 ± 2.394	5.43 ± 3.558	0.051
ALF			
*n*	29	30	
$S_{pO_{2}}$ mean	97.1 ± 2.3	96.3 ± 4.0	0.366
$S_{pO_{2}}$ entropy	0.155 ± 0.212	0.189 ± 0.234	0.556
δ	−0.00451 ± 0.199	−0.206 ± 0.236	0.778
SOFA	6.93 ± 3.954	7.73 ± 4.417	0.466
Cirrhosis			
*n*	89	80	
$S_{pO_{2}}$ mean	97.2 ± 3.2	96.7 ± 2.8	0.245
$S_{pO_{2}}$ entropy	0.192 ± 0.257	0.210 ± 0.244	0.650
δ	−0.0369 ± 0.254	−0.0404 ± 0.232	0.926
SOFA	5.76 ± 3.624	8.80 ± 4.268	**<0.00001**
MELD	11.401 ± 11.992	21.127 ± 14.524	**<0.00001**

*P*‐values shown in bold indicate statistical significance. ALF, acute liver failure; δ, directional parenclitic deviation; MELD: Model for End‐stage Liver Disease; SOFA, Sequential Organ Failure Assessment; $S_{pO_{2}}$, oxygen saturation.

Across disease groups, δ values displayed directionality relative to the healthy reference line. As shown in Table 2, patients with sepsis and COPD had positive δ, indicating their $S_{pO_{2}}$ entropy and mean values fall below the expected healthy trajectory. In contrast, patients with ALF and cirrhosis exhibited negative mean δ, suggesting a pattern of higher‐than‐expected entropy at given saturation levels.

$S_{pO_{2}}$ entropy and SOFA scores were also significantly different between survival groups in the sepsis group, with non‐survivors showing reduced entropy (*P* = 0.045) and higher SOFA score (*P* < 0.001). No significant difference was observed in the δ of COPD, ALF or cirrhosis survival groups. However, in cirrhosis, both SOFA (*P* < 0.00001) and MELD scores (*P* < 0.00001) were significantly higher in non‐survivors, consistent with expected disease severity trends.

### 3.3 Schematic interpretation of δ positioning across groups

**FIGURE 3:**
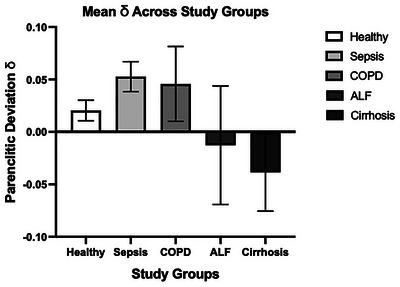
Mean parenclitic deviation (δ) across healthy and clinical groups. Bars represent group‐level means ± 95% confidence interval. δ quantifies the deviation of each patient's $S_{pO_{2}}$ entropy–mean relationship from the healthy reference pattern.

**FIGURE 4:**
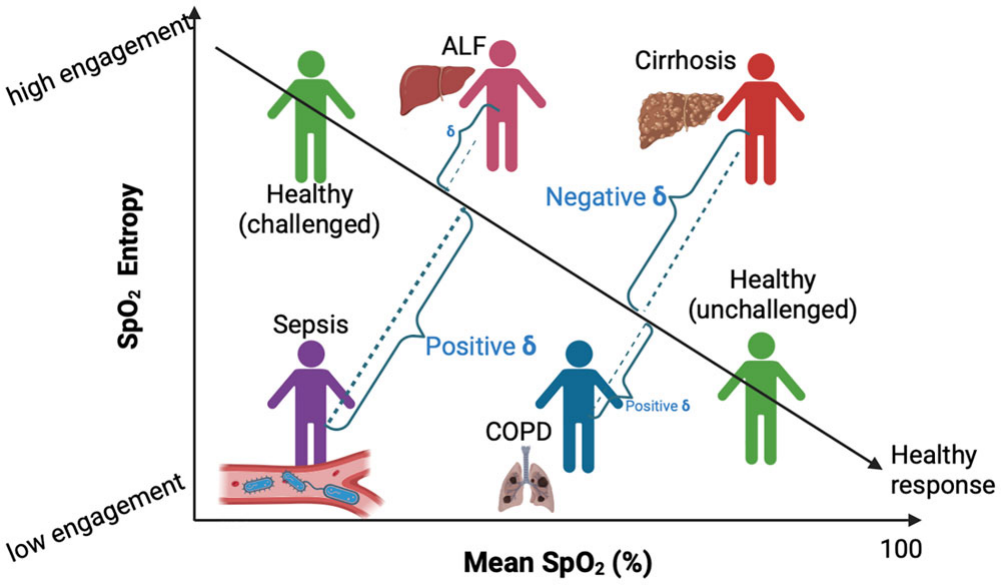
Schematic representation of disease group positioning relative to the healthy reference relationship between $S_{pO_{2}}$ entropy and mean $S_{pO_{2}}$. The black line represents the expected response in healthy individuals under varying levels of physiological challenge (hypoxic levels). Parenclitic deviation (δ) captures the shortest perpendicular distance from each group's observed position to the healthy reference line. Sepsis and COPD lie below the reference line with positive δ, indicating lower‐than‐expected entropy for a given mean $S_{pO_{2}}$. In contrast, cirrhosis and ALF lie above the line with negative δ, suggesting higher‐than‐expected entropy. These directional deviations provide insight into the system's regulatory engagement beyond entropy magnitude alone. (This is a visual schematic representation; δ in this figure are not according to scale).

To visualise this pattern, Figure 3 displays the average δ values for each group, showing that sepsis and COPD lie below the reference trajectory (positive δ), while cirrhosis and ALF lie above it (negative δ). This directional framing is further illustrated schematically in Figure 4, where cirrhosis appears in a zone of ‘over‐engagement’, exhibiting higher entropy than expected given relatively normal oxygenation. This is a notable deviation from healthy physiological behaviour, where entropy would normally increase in response to stress (e.g., hypoxia), but not in stable oxygenation environments. Conversely, patients with sepsis and COPD may be demonstrating under‐engagement, with reduced entropy despite moderate or low oxygenation. The raw data used to generate Figure 3 are provided in Supporting information, Supplementary Material 1.

### 3.4 Prognostic value of $S_{pO_{2}}$ entropy and parenclitic deviation at ICU

Table 3 summarises univariate Cox regression analyses for 30‐day ICU survival. In the sepsis group, $S_{pO_{2}}$ mean (*P *= 0.029), $S_{pO_{2}}$ entropy (*P *= 0.05), SOFA score (*P *< 0.001) and δ (*P *< 0.001) significantly predicted mortality. Specifically, each 1‐unit increase in SOFA score increased the hazard of death by 25% (hazard ratio = 1.249), and each standard deviation increase in δ was associated with more than double the risk of death (hazard ratio = 2.202). Conversely, higher mean $S_{pO_{2}}$ (hazard ratio = 0.004) and $S_{pO_{2}}$ entropy (hazard ratio = 0.180) were protective, reducing the risk of ICU 30‐day mortality. In the COPD, ALF and cirrhosis groups, none of the OSV metrics (mean, entropy, δ) were significantly associated with mortality.

**TABLE 3:** Univariate Cox regression analysis of 30‐day ICU mortality across disease groups.

	β	Hazard ratio (95% CI)	*P*
Sepsis (*n* = 164)			
$S_{pO_{2}}$ mean	−5.545	0.004 (0–0.568)	**0.029**
$S_{pO_{2}}$ entropy	−1.715	0.180 (0.032–0.999)	**0.050**
SOFA	0.222	1.249 (1.15–1.357)	**<0.001**
Δ	0.789	2.202 (1.615–3.002)	**<0.001**
COPD (*n* = 58)			
$S_{pO_{2}}$ mean	0.037	1.038 (0.904–1.191)	0.598
$S_{pO_{2}}$ entropy	1.130	3.096 (0.148–64.918)	0.467
SOFA	0.125	1.133 (0.983–1.305)	0.085
δ	−0.211	0.810 (0.544–1.205)	0.298
ALF (*n* = 59)			
$S_{pO_{2}}$ mean	−0.238	0.788 (0.541–1.148)	0.215
$S_{pO_{2}}$ entropy	−0.093	0.911 (0.655–1.267)	0.579
SOFA	−0.046	0.955 (0.869–1.049)	0.334
δ	−0.158	1.171 (0.819–1.676)	0.387
Cirrhosis (*n* = 169)			
$S_{pO_{2}}$ mean	−0.029	0.972 (0.911–1.036)	0.380
$S_{pO_{2}}$ entropy	0.297	1.346 (0.580–3.144)	0.489
MELD	0.016	1.016 (0.993–1.039)	0.179
δ	−0.060	0.942 (0.763–1.163)	0.577

*P*‐values shown in bold indicate statistical significance. ALF, acute liver failure; δ, directional parenclitic deviation; MELD: Model for End‐stage Liver Disease; SOFA, Sequential Organ Failure Assessment; $S_{pO_{2}}$, oxygen saturation.

To assess whether the predictive value of δ in the sepsis group depends on the severity of disease or ventilation status (i.e., mechanical ventilation during physiological recording), multivariate Cox regression analysis was employed. In the sepsis cohort, two multivariate Cox models were assessed (Table 4). In Model 1, which included ventilation (*P *= 0.002), SOFA score (*P *< 0.001) and δ (*P *< 0.001), all variables were significant mortality predictors. Mechanical ventilation (hazard ratio = 2.989), SOFA (hazard ratio = 1.194) and δ (hazard ratio = 1.794) each independently increased the risk of 30‐day death. In Model 2, where mean $S_{pO_{2}}$, $S_{pO_{2}}$ entropy, SOFA and ventilation were included, both mean $S_{pO_{2}}$ (hazard ratio = 0.872) and $S_{pO_{2}}$ entropy (hazard ratio = 0.001) were significant (*P *= 0.002 and *P *= 0.027, respectively) and protective, while SOFA and ventilation remained significant risk factors. This suggests that δ provides survival information for sepsis independent from both conventional severity scores (e.g., SOFA) and static oxygenation indices (e.g., mean $S_{pO_{2}}$) as shown in multivariate analysis in Table 4. When we compared Model 1 and Model 2, both demonstrated a similar model fit. The areas under the receiver operating characteristic (ROC) curve (AUCs) were also very similar for Model 1 and Model 2 (AUC [95% confidence interval]: 0.802 [0.713–0.891] and 0.805 [0.717–0.893], for Model 1 and Model 2, respectively), indicating that both models discriminate between individuals who survived and those who did not with comparable accuracy.

**TABLE 4:** Multivariate Cox regression analysis of 30‐day ICU mortality for critically ill patients with sepsis.

	β	Hazard ratio (95% CI)	*P*
Model 1			
δ	0.584	1.794 (1.267–2.539)	**<0.001**
SOFA	0.177	1.194 (1.084–1.316)	**<0.001**
Mechanical ventilation	1.095	2.989 (1.516–5.891)	**0.002**
Model 2			
$S_{pO_{2}}$ mean	−0.137	0.872 (0.801–0.949)	**0.002**
$S_{pO_{2}}$ entropy	−6.864	0.001 (4.65E‐5 ‐0.465)	**0.027**
SOFA	0.181	1.199 (1.086–1.323)	**<0.001**
Mechanical ventilation	1.054	2.868 (1.398–5.884)	**0.004**

*P*‐values shown in bold indicate statistical significance. δ, directional parenclitic deviation; SOFA, Sequential Organ Failure Assessment; $S_{pO_{2}}$, oxygen saturation.

### 3.5 Methodological robustness and sensitivity analysis

**FIGURE 5:**
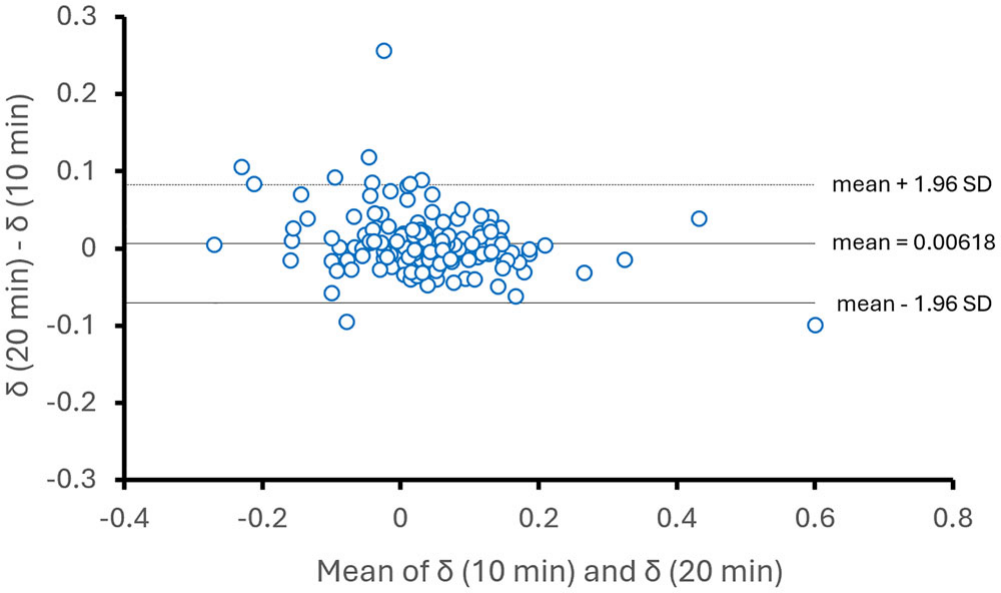
Bland–Altman plot of parenclitic deviation (δ) in 20 min vs. 10 min $S_{pO_{2}}$ signal duration. Bland–Altman analysis was performed on data from patients with sepsis to assess agreement between parenclitic deviation (δ) values calculated from 20‐min versus 10‐min $S_{pO_{2}}$ recordings. The analysis demonstrated no systematic bias associated with the shorter recording duration. Furthermore, only six participants had δ values lying outside the limits of agreement (mean difference ± 1.96 standard deviations), indicating good agreement between the two measurement durations.

**FIGURE 6:**
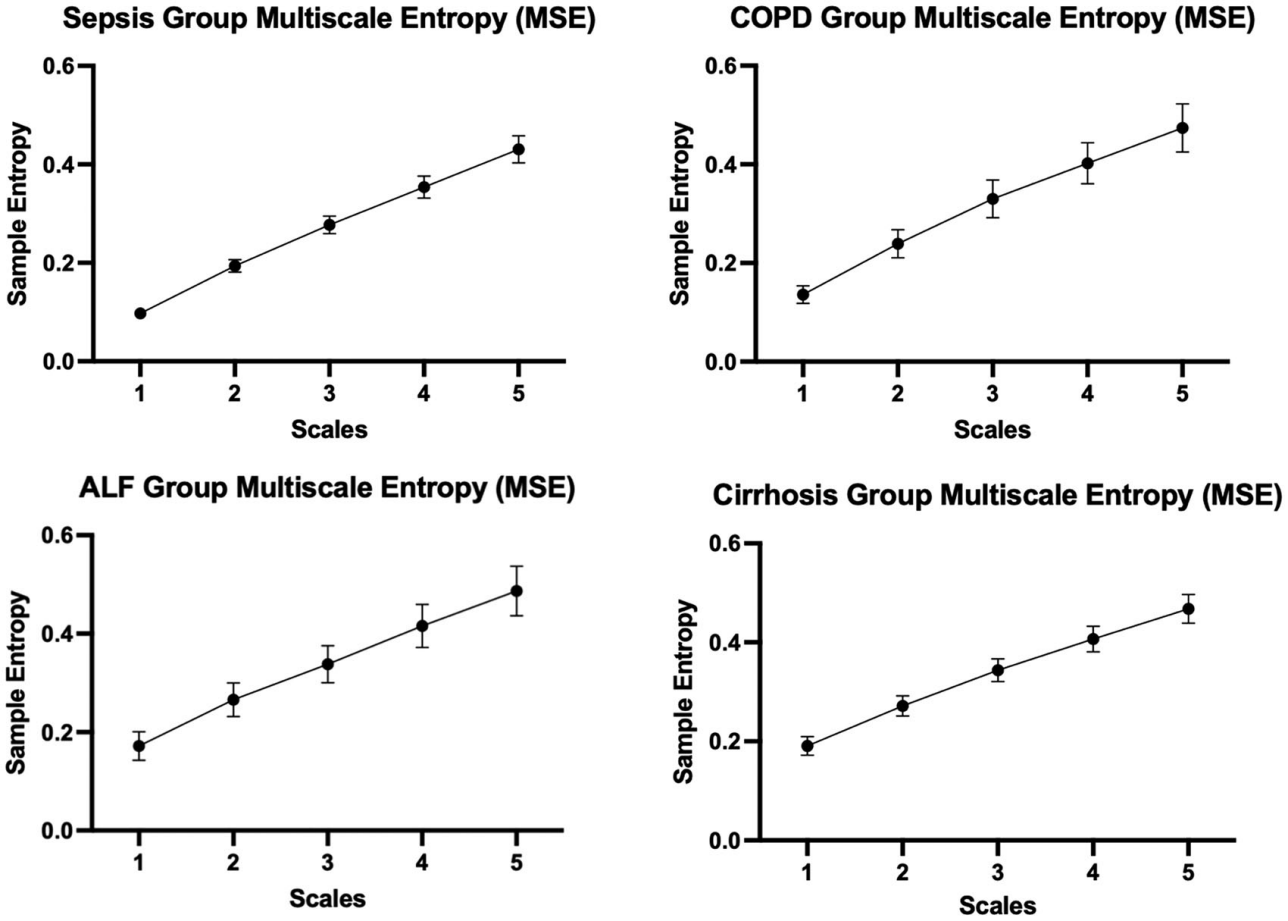
Multiscale entropy (MSE) profiles of $S_{pO_{2}}$ signals in the sepsis, COPD, ALF and cirrhosis groups. To quantify this complexity, multiscale entropy (MSE) analysis was applied across five time scales. Entropy increased consistently with scale in all clinical groups, indicating that $S_{pO_{2}}$ signals are complex and non‐random. According to Costa et al. (2002), random time‐series typically exhibit flat or decreasing entropy across scales, whereas physiological signals with meaningful complexity show an upward trend. The observed pattern confirms that $S_{pO_{2}}$ signals from ICU patients with these conditions retain intrinsic regulatory dynamics despite disease burden. Mean sample entropy values (±68% CI) are shown across five time scales. An upward trend in entropy with increasing scale is observed in all groups, consistent with the presence of structured, non‐random fluctuations in the $S_{pO_{2}}$ time‐series.

To assess whether the prognostic performance of $S_{pO_{2}}$ metrics and directional parenclitic deviation (δ) is dependent on time‐series duration, we repeated the Cox regression with only the first 10 min of each sepsis recording (1 Hz sampling, *n* = 164). As presented in Table 5, the hazard ratios and *P*‐values for mean $S_{pO_{2}}$, SOFA and δ closely mirrored the 20‐min analysis (Table 3), as strong predictors of 30‐day ICU mortality. In addition, a Bland–Altman analysis was performed on data from patients with sepsis to assess agreement between parenclitic deviation (δ) values calculated from 20‐min versus 10‐min $S_{pO_{2}}$ recordings (Figure 5). The analysis demonstrated no systematic bias associated with the shorter recording duration. Furthermore, only six participants had δ values lying outside the limits of agreement (mean difference ± 1.96 standard deviations) in the Bland–Altman plot, indicating good agreement between the two measurement durations. These findings indicate that the δ methodology is robust to the choice of time‐series duration, and even shorter recordings (e.g. 10 min) capture sufficient physiological information for prognostic interpretations. We did not assess the effect of different sampling rates of $S_{pO_{2}}$ recordings on entropy or δ in the present proof‐of‐concept study. This will require future analyses using different $S_{pO_{2}}$ measurement protocols in a more comprehensive study. However, we did assess the effect of scaling using coarse‐graining of the $S_{pO_{2}}$ signal on sample entropy (SampEn), also referred to as MSE analysis. The results, presented in Figure 6, indicate that entropy increased consistently with scale across all clinical groups. This finding suggests that $S_{pO_{2}}$ signals are complex and non‐random in the clinical settings studied, and that changes in entropy are not due to randomness but rather reflect increased signal complexity (Costa et al., 2002).

**TABLE 5:** Univariate Cox regression analysis of 30‐day ICU mortality in patients with sepsis.

	β	Hazard ratio (95% CI)	*P*
$S_{pO_{2}}$ **mean**	−5.687	0.003 (0.000–0.516)	**0.027**
$S_{pO_{2}}$ **entropy**	−0.118	0.888 (0.827–0.954)	**0.001**
**SOFA**	0.222	1.249 (1.150–1.357)	**<0.001**
δ	0.755	2.127 (1.569–2.884)	**<0.001**

10‐min $S_{pO_{2}}$ time‐series are used for calculation of $S_{pO_{2}}$ mean, sample entropy and δ.

## 4 DISCUSSION

### 4.1 Overview

This study developed a methodological framework for interpreting $S_{pO_{2}}$ variability by normalising $S_{pO_{2}}$ entropy relative to $S_{pO_{2}}$ mean and using directional parenclitic deviation (δ). The primary hypothesis that critically ill patients would demonstrate systematic deviations from the healthy entropy–mean $S_{pO_{2}}$ relationship, and that the direction and magnitude of this deviation (δ) would reflect distinct patterns of physiological dysregulation across disease types, is accepted.

### 4.2 Interpretation by disease groups

#### 4.2.1 Overview of the critical illnesses examined

Sepsis represents a life‐threatening dysregulation of the host response to infection (Singer et al., 2016), marked by microcirculatory dysfunction that contributes to reduced oxygen supply (Arora et al., 2023). Evidence suggests that the systemic response to sepsis is associated with a blunted response to physiological challenges (Gholami et al., 2012) and abnormal $S_{pO_{2}}$ dynamics (Gheorghita et al., 2022).

COPD is characterised by persistent airflow limitation and systemic inflammation that disrupt gas exchange profoundly, leading to ventilation–perfusion mismatch (MacNee, 2006). Over time, central respiratory control undergoes adaptive changes like blunted chemosensitivity to CO_2_ and hypoxic ventilatory drive, combined with mechanical constraints like dynamic hyperinflation, reducing the responsiveness of the respiratory system to fluctuating oxygen demands (Csoma et al., 2022; Jacono, 2013).

The liver serves as an important hub in the global physiological network, and liver failure is associated with increased risk of multi‐organ dysfunction, failure and mortality (Oyelade et al., 2024a). ALF is rapid loss of liver function in a patient without pre‐existing chronic liver disease. ALF is a life‐threatening disease, requiring intensive physiological support to maintain homeostasis (Oyelade et al., 2024b). Cirrhosis is a chronic disease caused by prolonged liver damage and is associated with systemic decompensation, including portal hypertension and multi‐organ network disruption (Ginès et al., 2012; Tan et al., 2020). Increased intrahepatic resistance from fibrosis and sinusoidal remodelling elevates portal pressure, leading to hepatic hypoperfusion and systemic consequences, including systemic and pulmonary vasodilation, cardiopulmonary dysfunction through cirrhotic cardiomyopathy and hepatopulmonary syndrome (HPS), which together disturb oxygen exchange and regulation (Jagdish et al., 2023; Møller & Bendtsen, 2015).

#### 4.2.2 Interpretation of findings

As anticipated, patients with sepsis and COPD exhibited positive δ, indicating blunted physiological engagement or reduced adaptive capacity. This is especially notable in sepsis, where δ was significantly higher in non‐survivors and was associated with more than double the risk of death. In sepsis, this pattern is mechanistically supported by the disease's hallmark endothelial dysfunction and microvascular dysfunction, which impair oxygen extraction and results in tissue hypoxia (Ince et al., 2016). The reduced $S_{pO_{2}}$ variability might be due to blunted compensatory mechanisms, such as tachycardia and increased ventilation in response to hypoxia, as there is evidence of end‐organ hypo‐responsiveness as well as impaired central autonomic regulation in sepsis (Gholami et al., 2012; Eftekhari et al., 2020). Non‐survivors had significantly higher δ, reflecting a more pronounced entropy deficit and a lack of system‐level complexity needed to adapt to hypoxic stress. Importantly, the predictive power of δ remained independent of SOFA score, suggesting that its ability to capture dynamic physiological dysfunction is not accounted for by static organ failure metrics.

In the COPD group, the consistently positive δ values suggested reduced respiratory system complexity despite chronic hypoxia. This finding is consistent with established pathophysiology, where fixed airflow obstruction and hyperinflation limit tidal volume adjustments, thereby suppressing signal variability (Feijani et al., 2025). This reflects impaired central respiratory control, a feature of advanced COPD. These findings align with the literature showing that hypercapnic COPD patients often tolerate elevated CO_2_ levels and exhibit minimal compensatory responses, a physiological compromise aimed at preserving respiratory muscle function (Mathews et al., 2020). In this context, δ may provide non‐invasive evidence of under‐engaged regulatory dynamics, suggesting that entropy suppression may be a measurable sign of chronic cardiorespiratory adaptation failure. Although δ did not distinguish survivors from non‐survivors in the COPD group, its consistency across individuals indicates potential utility as a supplementary marker for monitoring adaptive capacity in chronic respiratory conditions.

In contrast, the cirrhosis group demonstrated a novel and unexpected pattern, which showed negative δ values with entropy exceeding expected levels despite relatively preserved $S_{pO_{2}}$. This suggests increased signal complexity even in the absence of strong hypoxic challenge, potentially reflecting pathological overactivation or dysregulation. One plausible hypothetical explanation consistent with prior physiological literature, is the presence of HPS in cirrhosis (Raevens et al., 2022). HPS is a condition involving abnormally dilated pulmonary capillaries and the formation of intrapulmonary arteriovenous shunts, which together disrupt gas exchange; the intrapulmonary vascular dilations lead to ventilation–perfusion mismatch and limit oxygen diffusion (Qasim et al., 2024). Mixed perfusion states and compensatory dynamics may lead to elevated $S_{pO_{2}}$ entropy, even under seemingly stable inspired oxygen conditions, resulting in higher‐than‐normal entropy values and thus a negative δ. However, other mechanisms may also contribute, including autonomic dysfunction, peripheral vasodilation, and systemic endothelial activation associated with cirrhosis (Møller & Bendtsen, 2015). This counterintuitive pattern highlights the complexity of $S_{pO_{2}}$ variability in cirrhosis and underscores the importance of contextualising entropy within disease‐specific physiology. These findings challenge conventional interpretations of $S_{pO_{2}}$ variability analysis and highlight the need for contextualised, disease‐specific analyses. While the interpretation of negative δ and its association with HPS in critically ill patients with cirrhosis remains speculative, it warrants further investigation.

Currently, the methodology for diagnosing HPS relies on advanced invasive imaging techniques (e.g., contrast‐enhanced transthoracic echocardiography), which are not suitable for routine assessment (Forde et al., 2018). We hypothesised that increased $S_{pO_{2}}$ entropy relative to the mean $S_{pO_{2}}$ in patients with cirrhosis might reflect increased randomness (i.e., noise) rather than true physiological signal complexity. To explore this, we applied MSE analysis and found that $S_{pO_{2}}$ signals in cirrhotic patients exhibit genuine complexity (see Figure 6). This suggests that the elevated $S_{pO_{2}}$ entropy is not due to measurement noise or random fluctuations (Figure 6).

Moreover, we also speculated that the shift of δ toward negative values is related to liver failure itself, rather than being a complication of decompensated cirrhosis. To address this query, we included data from patients with ALF. The ALF group also showed slightly negative δ values, indicating a modest increase in entropy relative to $S_{pO_{2}}$. While this may reflect some degree of physiological dysregulation, it appears less pronounced and possibly more transient than the pattern observed in cirrhosis. This contrast may suggest that δ has potential as a marker to distinguish between acute and chronic liver failure pathophysiology, with negative δ possibly reflecting a chronic mechanism such as HPS, which is not typically observed in ALF. While these findings are intriguing, further studies are necessary to clarify the true interpretation of negative δ in critically ill patients with cirrhosis.

The broader healthy group used for comparison showed mildly positive δ. This is not unexpected, given that the healthy individuals in this study were not identical to the carefully screened, young and physically fit volunteers who comprised the hypoxia‐challenged reference dataset. Instead, the broader healthy cohort included a wider age range and more heterogeneous physiological baselines, which may naturally introduce slight reductions in regulatory efficiency. This highlights that the healthy reference group is not meant to represent a universal standard, but rather an idealised benchmark of optimal physiological regulation. Deviations from this benchmark, whether due to illness, undiagnosed clinical conditions or natural variation such as ageing, can be meaningfully interpreted.

### 4.3 Physiological implications

The increased deviation of $S_{pO_{2}}$ entropy from the reference line observed in sepsis and COPD is consistent with a reduction in the complexity of physiological signals in response to pathophysiological challenge. This phenomenon aligns with the de‐complexification hypothesis, described by Goldberger and colleagues over the past decades as a defining characteristic of critical illness (Goldberger, 1996). Our proposed metrics offer an alternative approach that addresses several limitations of traditional measures, such as the use of entropy as a standalone metric. While the present study focuses on refining the interpretation of $S_{pO_{2}}$ variability in the context of critical care, our approach is grounded in statistical insight rather than an exploration of the underlying physiological mechanisms governing tissue oxygenation. First, oxygen saturation signals are not direct measures of tissue oxygenation and are influenced by multiple physiological variables affecting the oxyhaemoglobin dissociation (Hill) curve, including pH, 2,3‐diphosphoglycerate and temperature. Consequently, it would be overly simplistic to assume that parenclitic deviation calculations provide detailed mechanistic insight into cardiorespiratory control. However, a key advantage of this method is its network‐level perspective rather than a focus on isolated physiological components. This approach may yield insights into the integrity of physiological networks that cannot be directly assessed using conventional techniques such as arterial blood gas analysis or biochemical measurements. Our findings are consistent with previous reports suggesting that pattern analysis of $S_{pO_{2}}$ fluctuations can provide information about physiological network integrity using information‐theoretic methods (e.g., transfer entropy) (Jiang et al., 2021). Importantly, this framework is not limited to $S_{pO_{2}}$ analysis. Foundational work in HRV research has shown that neglecting the relationship between HRV and basal heart rate (largely due to limited understanding of the biophysics of pacemaker currents) has led to frequent misinterpretations of HRV in the biomedical literature (Monfredi et al., 2014). Consequently, both mechanistic investigations and network‐based approaches should be considered when developing and interpreting physiological biomarkers for clinical practice.

### 4.4 Limitation and potential sources of bias

Several limitations should be considered when interpreting the findings of this study.

First, the healthy reference dataset is limited in size and demographic diversity. The sample consisted of only 12 young, healthy participants, male‐dominant, without stratification by age or sex. Although adequate for proof‐of‐concept analysis, this lack of diversity may limit generalisability when applying the reference model to broader patient populations. Thus, the results from the reference population in this study should not be used for clinical application; they serve solely as a proof‐of‐concept to advance metrics for the interpretation of $S_{pO_{2}}$ entropy analysis.

Second, the patient data were obtained from the MIMIC‐III database, which consists of ICU admissions from well‐resourced teaching hospitals, specifically Beth Israel Deaconess Medical Centre in Boston, Massachusetts (Johnson et al., 2016). This introduces potential selection bias, as the population may not be representative of broader clinical settings, such as in low‐resource or community‐level hospitals. Additionally, although this study focuses on critically ill individuals, it is important to acknowledge that not all patients with cirrhosis require ICU‐level care. The dataset may also include acute‐on‐chronic liver failure (ACLF), which is a severe subset of cirrhosis with multi‐organ involvement and may not reflect the full spectrum of chronic liver failure.

Another potential bias is related to the signal selection process. To ensure data quality, 20‐min continuous $S_{pO_{2}}$ recordings were included. This criterion may unintentionally exclude individuals with agitation or severe instability, as movement and frequent intervention can disrupt waveform integrity and result in poor‐quality recordings. Consequently, many patients were excluded due to poor‐quality data, which may introduce selection bias. In this proof‐of‐concept study, our primary focus was on obtaining noise‐free data to investigate the concept of normalising $S_{pO_{2}}$ entropy by its mean, without introducing the additional complexity of handling noisy signals. Future studies could adopt less stringent exclusion criteria to evaluate the prognostic value of this novel metric in patient populations that more accurately reflect real‐world clinical environments.

Due to limitations in sample size and the availability of longer clean signals, only 10‐min $S_{pO_{2}}$ time‐series were used in the ALF group. While this is a limitation, it is known that SampEn is largely independent of the length of the time‐series (Richman and Moorman, 2000). Moreover, we compared 10‐min and 20‐min $S_{pO_{2}}$ signals in the sepsis group (Table 5 and Figure 1a) and observed acceptable agreement between the two measurement durations for parenclitic deviation (δ). This suggests greater clinical flexibility in studying δ, as it is more convenient in clinical practice to obtain shorter, noise‐free time‐series.

In this study, we did not have access to high‐resolution haemodynamic or pulmonary function data, nor to detailed information on drugs (e.g. vasopressor) use, all of which may influence clinical outcomes. These factors should be considered in future investigations.

### 4.5 Future directions

To enhance this framework's generalisability, future studies should recruit a more diverse and demographically representative healthy reference population, with balanced inclusion across age groups, sex and other relevant physiological factors. The present study also relied on ICU data from a single tertiary care centre in the United States. Expanding the analysis to include patient data from multiple healthcare systems, particularly from community and lower‐resource settings, will help validate the robustness and adaptability of directional parenclitic deviation (δ) across diverse clinical environments. Additionally, future patient‐level analyses should aim to include cases from earlier stages of disease severity, particularly for cirrhosis, where non‐ICU data may reflect more typical chronic profiles.

From a statistical standpoint, while this study focused on widely used severity indices such as SOFA and MELD scores, future multivariate models could be strengthened by incorporating additional covariates including comorbidity burden, medication history (e.g., use of beta‐blockers or corticosteroids), and pre‐admission functional status (Singer et al., 2017; Medina‐Mirapeix et al., 2022; Hayward & Weersink, 2020). This would provide a more holistic view of how δ interacts with complex clinical backgrounds. Moreover, the use of alternative non‐parametric approaches may help address potential deviations from normality in entropy‐related measures.

Looking ahead, parenclitic deviation could potentially act as a non‐invasive, dynamic marker of dysregulation in different disease contexts. For example, in cirrhosis, δ might serve as a surrogate indicator of HPS, offering a potential non‐invasive alternative to imaging‐based diagnostic tools such as contrast‐enhanced echocardiography or intravenous injection of radioactive isotopes Technetium‐99m‐labelled albumin macroaggregates, which are costly, involve radiation exposure (∼2 mSv, equivalent to 100 chest X‐rays) and offer only a single time‐point snapshot of the disease (Grilo‐Bensusan & Pascasio‐Acevedo, 2016; Gargani & Picano, 2015). If validated, δ could potentially provide a safer, repeatable alternative to monitor early pulmonary vascular abnormalities over time.

Beyond its prognostic use, δ can be integrated within a broader framework of physiological time‐series complexity analysis, such as transfer entropy and metrics like HRV and $S_{pO_{2}}$/$F_{iO_{2}}$ ratio, to potentially serve as a multi‐model biomarker (Bodénes et al., 2022). The calculation of δ is computationally lightweight, as it requires only the mean and entropy over short $S_{pO_{2}}$ segments. Since it relies on routinely collected pulse oximetry data, δ represents a low‐cost and non‐invasive approach for evaluating physiological control.

Additionally, in ICU settings, where oxygen supplementation is often liberal and $S_{pO_{2}}$ is maintained within high‐normal ranges (96–100%) despite evidence of oxygen toxicity, δ may also offer clinical value in guiding oxygen titration (Capellier et al., 2023). In conditions like sepsis and COPD, where dynamic physiological control is often impaired, δ could be used as a real‐time signal to evaluate whether oxygen delivery strategies are physiologically appropriate, rather than purely target‐based. Thus, δ may eventually complement or inform personalised oxygen therapy protocols in critical care.

Regardless of potential applications, future studies should also examine sensitivity to noise, recording sampling rates, and imputation and preprocessing choices that arise when using inherently noisy clinical data. Such analyses will help assess the feasibility of these methodologies in real‐time clinical scenarios.

### 4.6 Conclusion

This study demonstrated that contextualising OSV through parenclitic deviation (δ) from a healthy $S_{pO_{2}}$ mean–entropy reference provides new insight for the physiological regulation of $S_{pO_{2}}$ dynamics in critical illness. In both sepsis and COPD, positive δ values indicate lower‐than‐expected entropy for a given $S_{pO_{2}}$, reflecting impaired adaptive engagement of respiratory and vascular control mechanisms. Conversely, patients with cirrhosis exhibited negative δ, representing unexpectedly high entropy despite relatively preserved oxygenation. This may reflect pathological processes unique to this population and warrants further investigation. In addition, in the sepsis group δ was significantly higher in non‐survivors than survivors and was associated with a higher risk of death. These findings support the use of parenclitic deviation as a meaningful, disease‐specific marker that complements traditional static metrics, offering a novel framework for integrating complexity analysis into critical care monitoring and individualised risk assessment.

## AUTHOR CONTRIBUTIONS

Conception or design of the work: Shuyang Iris Feng, Tope Oyelade, Ali R. Mani Acquisition, analysis, or interpretation of data for the work: Shuyang Iris Feng, Tope Oyelade, Mudra Ko, Yunkai Zhang, Watjana Lilaonitkul, Thomas B. Williams, Joseph T. Costello, Ali R. Mani Drafting of the work or revising it critically for important intellectual content: Shuyang Iris Feng, Tope Oyelade, Mudra Ko, Yunkai Zhang, Watjana Lilaonitkul, Thomas B. Williams, Joseph T. Costello, Ali R. Mani. All authors have read and approved the final version of this manuscript and agree to be accountable for all aspects of the work in ensuring that questions related to the accuracy or integrity of any part of the work are appropriately investigated and resolved. All persons designated as authors qualify for authorship, and all those who qualify for authorship are listed.

## CONFLICT OF INTEREST

The authors declare that the research was conducted in the absence of any commercial or financial relationships that could be construed as a potential conflict of interest.

## Supporting information



Supplementary Material 1. The raw data used to generate Figure 3 showing individual δ values for Healthy individuals as well as patients with Sepsis, COPD, ALF and Cirrhosis. This supplementary material is available on Figshare: https://doi.org/10.6084/m9.figshare.31073158

## Data Availability

Data will be made available upon reasonable request.
